# Simple framework for real-time forecast in a data-limited situation: the Zika virus (ZIKV) outbreaks in Brazil from 2015 to 2016 as an example

**DOI:** 10.1186/s13071-019-3602-9

**Published:** 2019-07-12

**Authors:** Shi Zhao, Salihu S. Musa, Hao Fu, Daihai He, Jing Qin

**Affiliations:** 1 School of Nursing, Hong Kong Polytechnic University, Hong Kong, China; 2 Department of Applied Mathematics, Hong Kong Polytechnic University, Hong Kong, China; 3 Department of Crop Science and Technology, College of Agriculture, South China Agricultural University, Guangzhou, China

**Keywords:** Zika virus, Brazil, Modeling analysis, Reproduction number, Epidemic size, Spatial heterogeneity

## Abstract

**Background:**

In 2015–2016, Zika virus (ZIKV) caused serious epidemics in Brazil. The key epidemiological parameters and spatial heterogeneity of ZIKV epidemics in different states in Brazil remain unclear. Early prediction of the final epidemic (or outbreak) size for ZIKV outbreaks is crucial for public health decision-making and mitigation planning. We investigated the spatial heterogeneity in the epidemiological features of ZIKV across eight different Brazilian states by using simple non-linear growth models.

**Results:**

We fitted three different models to the weekly reported ZIKV cases in eight different states and obtained an *R*^2^ larger than 0.995. The estimated average values of basic reproduction numbers from different states varied from 2.07 to 3.41, with a mean of 2.77. The estimated turning points of the epidemics also varied across different states. The estimation of turning points nevertheless is stable and real-time. The forecast of the final epidemic size (attack rate) is reasonably accurate, shortly after the turning point. The knowledge of the epidemic turning point is crucial for accurate real-time projection of the outbreak.

**Conclusions:**

Our simple models fitted the epidemic reasonably well and thus revealed the spatial heterogeneity in the epidemiological features across Brazilian states. The knowledge of the epidemic turning point is crucial for real-time projection of the outbreak size. Our real-time estimation framework is able to yield a reliable prediction of the final epidemic size.

**Electronic supplementary material:**

The online version of this article (10.1186/s13071-019-3602-9) contains supplementary material, which is available to authorized users.

## Background

Zika virus (ZIKV) was first identified in the Zika Forest of Uganda in 1947 [1]. Later it was found to spread in the human populations in Nigeria [2, 3]. ZIKV is an arbovirus in the family of *Flaviviridae* and is transmitted through the bites of mosquito vectors (usually of *Aedes aegypti* mosquitoes) [4–7]. By 2007, ZIKV had escaped Africa to the Yap island in Micronesia, and it infected an estimated 75% of the local population [8]. In 2013, ZIKV reached French Polynesia and caused an infection attack rate of 49% [9, 10]. By 2015 it had invaded Brazil [10–12] and then quickly the many regions in South America [4, 13, 14]. Since 2015, other ZIKV transmission routes have also been found (materno-fetal, sexual transmission and *via* blood transfusion) [15–17], but these paths are uncommon and inefficient [12]. Up to the end of 2018, ZIKV infections had been reported in 86 countries (or regions) mainly in Oceania and the Americas [17]. In recent years, available scientific evidence and analysis strongly suggest that ZIKV could cause Guillain-Barré syndrome (GBS) [17–21]. ZIKV infection in pregnant women is also reported to be associated with, among other medical complications, microcephaly in their infants and sometimes even fetal deaths [17, 22–24]. Due to the lack of effective vaccines or medication, the World Health Organization (WHO) declared ZIKV as a public health emergency of international concern as of February 2016 [17].

In Brazil, samples of eight patients (with rash) tested at the Bahia State laboratory were positive for ZIKV by RT-PCR in epidemiological week (EW) 17 of 2015 [11, 25]. In EW 19 of 2015, Brazil authorities reported positive results for ZIKV by RT-PCR in samples taken from the States of Rio Grande and Bahia. This was the first report of locally-acquired ZIKV infection in Brazil [25]. The first wave of ZIKV hit northeastern Brazil in the first quarter of 2015 and started fading out since September, and was severely underreported since the mandatory ZIKV case notification only started in February 2016 [26]. The second wave of ZIKV swept Brazil between October 2015 and July 2016 [4, 27], followed by an increasing number of microcephaly infants across the whole country [28–30], as well as GBS cases [13]. This second wave in Brazil ended around July 2016, and even earlier for some of the states [25].

Modelling is widely used to study primary epidemic features and estimate the epidemiological parameters in the infectious disease outbreaks [10, 12, 14, 15, 31–37]. During an outbreak, the crucial epidemiological parameters include the reproduction number [32, 34, 38–40], final epidemic size [41, 42] and the turning time point [43–47]. The three parameters reflect the levels of infectivity, severity and the inflection time point of an epidemic, respectively. Knowledge of these epidemiological parameters summarize the temporal pattern of an epidemic and is helpful to understand the features of an outbreak. The real-time prediction of the epidemic final size is a procedure in which the estimates are valuable if achieved early. Moreover, the real-time estimation of the potential severity of an ongoing epidemic could be crucial for disease control and prevention policy-making [46, 48–52].

In this study, we were inspired by previous work [44, 46, 53] and adopted simple non-linear phenomenological models to study the epidemics in a data-limited situation. When a relatively new disease hits an under (or less) developed population, much public health related information is unknown during the outbreak, and only reported case time series are available. However, a quick estimate of the key epidemiological parameters and forecast on the trend is crucial for mitigation planning. We propose a framework for such a situation and use the ZIKV case time series in eight Brazilian states as an example. We study the power of simple models for real-time estimation in a data-limited situation. We forecast the final epidemic size in real-time. We reveal the spatial heterogeneity of epidemiological parameter estimates of the ZIKV epidemics across Brazil which should be useful in mitigation planning (or resource allocation).

## Methods

### Data

We obtained weekly reported ZIKV cases (both confirmed and suspected or suspected only) in eight Brazilian states between January 2015 to July 2016 from published literature [4]. These states include Acre, Bahia, Pernambuco, Espirito Santo, Parana, Rio Grande, Goiania City and Mato Grosso. According to the case definition by the WHO [54], a confirmed case must be first defined as a suspected case, and thus we follow previous work [10, 44] to use either the sum of confirmed and suspected (if they are available) or the suspected cases for analysis. Although Brazil started national wide mandatory ZIKV case notification on February 2016 [4, 26], many states with large-scale outbreak started local notification (reporting) on or after October 2015. The second large epidemic wave of ZIKV infections started in October 2015 [4, 27]. In this work, we use the ZIKV epidemic data on or after October 2015 for all eight states in Brazil for modelling.

We obtained the population data at the end of 2015 from the Brazilian Institute of Geography and Statistics [55].

### Mathematical models

We aimed to investigate the temporal patterns and transmission potential of ZIKV in eight Brazilian states over roughly the same period of time in 2015–2016. We adopted three different non-linear growth models to pinpoint the wave of ZIKV infections in each state. The three models are the three-parameter logistic growth model [56], the Gompertz growth model [57] and the Richards model [58], which are widely used to study S-shaped cumulative growth processes, e.g. epidemic curves [43, 44, 46, 47, 53, 59].

In this study, we denote the real (or theoretical) cumulative number of ZIKV cases of time (or day) *t* by *C*(*t*), and thus also *C*(*t*) represents the instantaneous epidemic size at time *t*. The three-parameter logistic growth model reads

1 $$ C\left( t \right) = \frac{K}{{1 + e^{{ - \gamma \left( {t - \tau } \right)}} }}. $$

The Gompertz growth model reads

2 $$ C\left( t \right) = Ke^{{ - e^{{ - \gamma \left( {t - \tau } \right)}} }} . $$

The Richards model reads

3 $$ C\left( t \right) = \frac{K}{{\left[ {1 + \alpha e^{{ - \alpha \gamma \left( {t - \tau } \right)}} } \right]^{{\left( {1/\alpha } \right)}} }}. $$

In Eqns (1–3), *K* is the maximum cumulative case number or the final epidemic size over the single wave of an outbreak, *γ* is the intrinsic per capita growth rate of the infected population, and *τ* is the unique inflection time point. For the Richards model in Eqn (3), term *α* is the exponent of deviation of the cumulative S-shaped ZIKV epidemic curve for *C*(*t*). Especially, when *α* = 1, the Richards model Eqn (3) becomes the logistic model in Eqn (1). Different from the logistic model in Eqn (1), the Richards model is no longer symmetrical about the point of inflection (*τ*) when *α* ≠ 1. More similarities and difference among the three growth models are summarized in Additional file 1: Text S1. Unlike the standard “susceptible-infectious-recovered” (SIR) compartmental models commonly used to study the transmission of diseases [12, 14, 36], these growth models consider the cumulative cases with saturation in the growth rate as the signs of progress of epidemics. The extrinsic growth rate does not steadily decline but rather increases to a maximum (i.e. a saturated level) before steadily declining to zero.

The turning point or inflection point, *τ*, is defined as the time point when the sign change in the rate of cases accumulation occurs, i.e. changes from increasing to decreasing or *vice versa*. Hence, *τ* is the moment at which the daily (or weekly) incidence trajectory begins to decline, which means the extrinsic growth rate reaches its maximum. The turning point indicates the beginning of an epidemic phase changing from the acceleration to deceleration.

The model parameters *K*, *γ* and *τ* are of epidemiological importance. These parameters can be estimated by fitting the growth models to the epidemic data of the ZIKV outbreak. We adopted the standard non-linear least squares (NLS) approach for model fitting and parameter estimation. Thus, the real cumulative case number at time *t*, *C*(*t*), is assumed to follow a normal distribution with a mean of the reported cumulative number of cases and an unknown but constant variance [43, 44, 46, 47]. A *P*-value of < 0.05 is regarded as statistically significant, and the 95% confidence intervals (CI) for all unknown parameters are estimated.

**Fig. 1:**
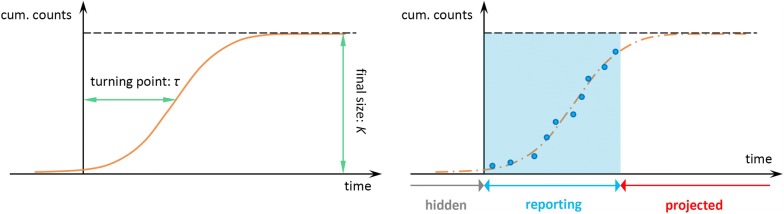
The illustration diagram of the modelling framework. The (solid and dashed) orange lines are the theoretical growth curves from the growth models in Eqns 1–3. The blue dots are the reported cumulative (cum.) number of cases. The blue shading area represents the time period when the disease notification is ongoing, which is also the time period for the model fitting

The epidemic data in each state are fitted by all three growth models in Eqns (1–3) (see Fig. 1 for an illustration). We adopted the *R*^2^ to measure the goodness-of-fit of each model. Since the models have different numbers of unknown parameters, the Akaike information criterion (AIC) was used to evaluate model performance in terms of the trade-off between the goodness-of-fit and the model complexity. For each state, the model with the lowest AIC value was chosen for further evaluation on its potentials for the real-time estimation.

### Reproduction number

The reproduction number, *R*, is the average number of secondary infectious cases produced by one infectious case during a disease outbreak [40, 45, 60]. When a population is totally (i.e. 100%) susceptible, *R* becomes the basic reproduction number, *R*_*0*_ [39, 61]. When a disease reaches a place (or region) for the first time, the estimated *R* can therefore be treated as *R*_*0*_. Following previous studies [38, 40, 60, 62], the reproduction number (*R*) is given in Eqn (4).

4 $$ R = \frac{1}{{M\left( { - \gamma } \right)}} = \frac{1}{{\mathop \smallint \nolimits_{0}^{\infty } e^{ - \gamma \kappa } h\left( \kappa \right)   {\text{d}}\kappa }}. $$

Here, *γ* is the intrinsic per capita growth rate from the growth model in Eqns (1–3), and *κ* is the serial interval of the ZIKV infection. The serial interval (i.e. generation interval) is the average time interval from onset of one individual to the onset of another individual infected by him/her [44], or the time between successive cases in a chain of transmission [4, 38, 40, 62, 63]. The function *h*(∙) represents the probability distribution of the serial interval, *κ*. Hence, the function *M*(∙) is the Laplace transform of *h*(∙), specifically, *M*(∙) is known to statisticians as the moment generating function (MGF) of a probability distribution.

According to previous work [4], we set *h*(*κ*) to be a gamma distribution with a mean of 20.0 days and standard deviation (SD) of 7.4 days, the SDs of the mean and SD of *κ* are 2.3 and 1.3 days, respectively. Therefore, *R* can be estimated with the values of *γ* from models (1–3).

### Projection of the epidemic and real-time estimation

In each state, we chose the model attaining the lowest AIC and simulated it into the future to estimate the final size (*K*) of the outbreak. To evaluate the real-time forecast power, we repeated the fitting procedure starting from the full epidemic wave to a sequence of waves with the end week discarded. We denoted the start, the end and the turning pint of the outbreak by 0, *T* and *τ*, such that 0 < *τ* < *T*. In the fitting part, we used the all data from time 0 to *T* to fit the models and estimate parameters. For the real-time estimation, we used the data from time 0 to *T*_*1*_, where 0 < *T*_*1*_ < *T*, so that the model was fitted with an incomplete dataset. With initially *T*_*1*_ = *T*, we decreased *T*_*1*_ gradually from time *T* backwards until the model fitting diverged. We compared the real-time estimated final size (*K*) and the *K* estimate based on the complete dataset, and stopped the real-time estimation when the yielded confidence interval was too wide (e.g. including zero) to be useful at the end of epidemic period, i.e. *T*. We checked the parameter estimates and compared with the results from the full-data modelling to evaluate the analysis sensitivities as the measurement of the real-time estimating potential. The (real-time) estimates with the lowest three *T*_*1*_s were compared to the estimation based on the full dataset.

The knowledge of the turning point (*τ*) is crucial for real-time projection [44–46], but this information is usually not precisely available. Alternatively, for Zika disease, one may gain knowledge of *τ* from the mosquito vectors’ activities. Since the mosquito abundance in Brazil decreases around May each year [64], we attempted to project the final size (*K*) with *τ* fixed at three dates prior to May, namely the first days of February, March and April 2016. Similarly, for the real-time projection, we used the data from time 0 to *T*_*1*_, where 0 < *T*_*1*_ < *T*, to train the model. The growth model was fitted with the dataset from 0 to *T*_*1*_, and used to project *K* in real-time. We compared the real-time projection of *K* with the estimates based on full data to measure the forecast power (performance) of the model.

## Results

**Fig. 2:**
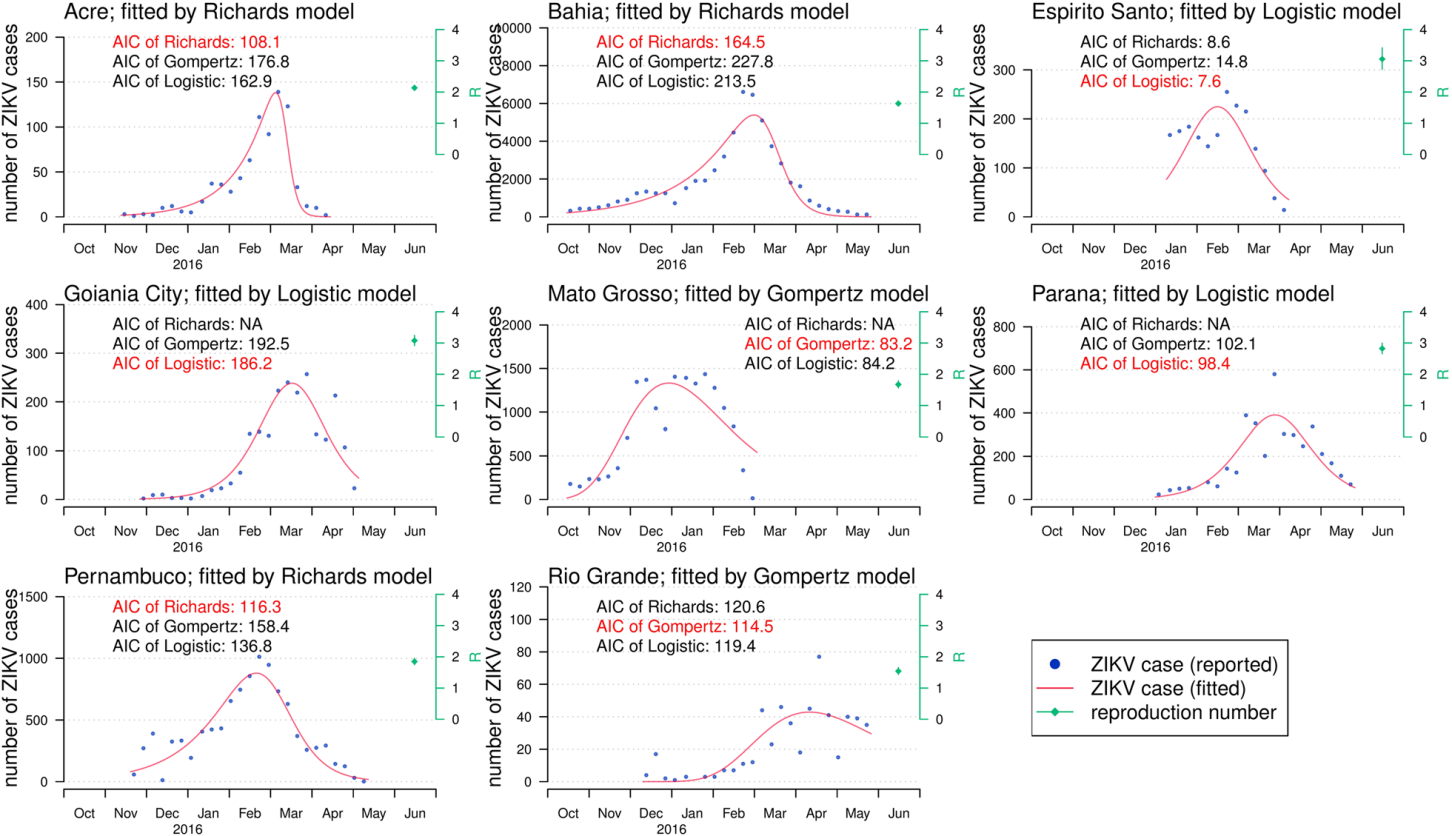
The fitting results of the ZIKV epidemics and the estimates of the reproduction number, *R*. The dots are the number of reported weekly ZIKV incidences, and the red curves are the fitted epidemic curves by the model with the lowest AIC (highlighted in red). The cyan diamond at the top-left corner of each panel is the reproduction number estimation, and the bar is the 95% CI

We fitted the three different models in Eqns (1–3) to the time series data of ZIKV incidences number from eight states in Brazil from October 2015 to May 2016. Figure 2 shows that the selected models can provide a good fit to the observations. All selected models achieved a level of *R*^2^ larger than 0.995. For each state, we selected the fitting results with the lowest value of AIC as the most suitable model (Table 1, Fig. 2). In particular, the Richards model is selected for Acre, Bahia, and Pernambuco; Gompertz model is selected for Mato Grosso and Rio Grande; and the logistic model is selected for Espirito Santo, Goiania City and Parana. The reproduction number, *R*, estimates vary from 1.54 to 3.07 for the eight different states (Table 1). We estimate *R* = 1.54 (95% CI: 1.43–1.65) in Rio Grande, and *R* = 3.07 (95% CI: 2.92–3.24) in Goiania City. The estimated dates of the turning points also vary from January to April of 2016, with 4 out of 8 states in March 2016. For the same state, the final size estimates from different models are roughly consistent, with the 95% CIs largely overlapping. The estimated final (epidemic) sizes, *K*, are also summarized in Table 1. We estimate the largest final outbreak size of 55,472 (95% CI: 54,683–56,260) in Bahia for the outbreak since October 2015, after the one epidemic wave in early 2015 [4, 10].

**Table 1:** Summary table of the model fitting and estimation results. The models results summarized here are also estimated by using the full epidemic dataset during the whole epidemic period. The models with the lowest AICs (for the same states) are considered as main results, which matches the results in Fig 2. The numbers in parentheses are the 95% CIs

State	Population	Model	Duration^a^	Epidemic notice period	Final size	Reproduction number	Turning point^b^	Turning date	*R* ^2^	AIC
Acre	803,513	Richards	148	26.11.2015–23.04.2016	783 (774–793)	2.13 (2.07–2.19)	111 (110–113)	17.03.2016 (15.03.2016–18.03.2016)	0.9995	108.1
Acre	803,513	Gompertz	148	26.11.2015–23.04.2016	925 (804–1045)	2.25 (1.82–2.76)	96 (91–100)	01.03.2016 (25.02.2016–05.03.2016)	0.9887	176.8
Acre	803,513	Logistic	148	26.11.2015–23.04.2016	858 (804–912)	3.45 (2.94–4.04)	101 (98–104)	07.03.2016 (03.03.2016–10.03.2016)	0.9934	162.9
Bahia	15,203,934	Richards	218	29.10.2015–04.06.2016	55,472 (54,683–56,260)	1.63 (1.58–1.68)	135 (132–139)	13.03.2016 (09.03.2016–16.03.2016)	0.9984	164.5
Bahia	15,203,934	Gompertz	218	29.10.2015–04.06.2016	59,773 (56,293–63,252)	1.89 (1.68–2.13)	113 (110–117)	20.02.2016 (16.02.2016–24.02.2016)	0.9885	227.8
Bahia	15,203,934	Logistic	218	29.10.2015–04.06.2016	58,896 (56,603–61,189)	2.21 (2.03–2.40)	121 (118–125)	27.02.2016 (24.02.2016–02.03.2016)	0.9922	213.5
Espirito Santo	3,929,911	Richards	84	23.01.2016–16.04.2016	2066 (1913–2219)	2.42 (1.78–3.25)	38 (32–44)	29.02.2016 (23.02.2016–07.03.2016)	0.9963	8.6
Espirito Santo	3,929,911	Gompertz	84	23.01.2016–16.04.2016	2293 (1993–2592)	2.11 (1.68–2.64)	30 (25–35)	22.02.2016 (16.02.2016–27.02.2016)	0.9940	14.8
Espirito Santo	3,929,911	Logistic	84	23.01.2016–16.04.2016	2132 (2020–2243)	3.05 (2.73–3.41)	35 (32–38)	27.02.2016 (24.02.2016–29.02.2016)	0.9960	7.6
Goiania City	6,610,681	Richards	155	10.12.2015–14.05.2016	na	na	na	na	na	na
Goiania City	6,610,681	Gompertz	155	10.12.2015–14.05.2016	2578 (2437–2718)	1.89 (1.79–2.00)	104 (102–106)	23.03.2016 (21.03.2016–25.03.2016)	0.9989	192.5
Goiania City	6,610,681	Logistic	155	10.12.2015–14.05.2016	2243 (2184–2303)	3.07 (2.92–3.24)	109 (108–111)	29.03.2016 (27.03.2016–30.03.2016)	0.9991	186.2
Mato Grosso	3,265,486	Richards	134	29.10.2015–12.03.2016	na	na	na	na	na	na
Mato Grosso	3,265,486	Gompertz	134	29.10.2015–12.03.2016	19,791 (18,147–21,435)	1.67 (1.56–1.79)	73 (69–76)	10.01.2016 (06.01.2016–14.01.2016)	0.9978	83.2
Mato Grosso	3,265,486	Logistic	134	29.10.2015–12.03.2016	17,165 (16,411–17,920)	2.47 (2.31–2.65)	79 (77–82)	17.01.2016 (14.01.2016–19.01.2016)	0.9974	84.2
Parana	11,163,018	Richards	141	14.01.2016–04.06.2016	na	na	na	na	na	na
Parana	11,163,018	Gompertz	141	14.01.2016–04.06.2016	4382 (4162–4602)	1.92 (1.80–2.04)	79 (77–81)	02.04.2016 (31.03.2016–02.04.2016)	0.9984	102.1
Parana	11,163,018	Logistic	141	14.01.2016–04.06.2016	4008 (3894–4123)	2.82 (2.66–2.99)	86 (84–87)	09.04.2016 (07.04.2016–11.04.2016)	0.9986	98.4
Pernambuco	9,345,173	Richards	169	03.12.2015–21.05.2016	9936 (9770–10,102)	1.85 (1.75–1.95)	90 (87–93)	02.03.2016 (29.02.2016–05.03.2016)	0.9990	116.3
Pernambuco	9,345,173	Gompertz	169	03.12.2015–21.05.2016	10,721 (10,168–11,273)	1.85 (1.69–2.01)	76 (73–79)	17.02.2016 (14.02.2016–21.02.2016)	0.9945	158.4
Pernambuco	9,345,173	Logistic	169	03.12.2015–21.05.2016	10,323 (10,070–10,576)	2.33 (2.21–2.45)	84 (82–86)	25.02.2016 (23.02.2016–27.02.2016)	0.9975	136.8
Rio Grande	11,247,972	Richards	162	24.12.2015–04.06.2016	595 (492–698)	1.98 (1.65–2.36)	124 (119–129)	26.04.2016 (21.04.2016–01.05.2016)	0.9962	120.6
Rio Grande	11,247,972	Gompertz	162	24.12.2015–04.06.2016	772 (653–890)	1.54 (1.43–1.65)	120 (112–128)	22.04.2016 (14.04.2016–30.04.2016)	0.9971	114.5
Rio Grande	11,247,972	Logistic	162	24.12.2015–04.06.2016	634 (575–693)	2.15 (2.00–2.32)	124 (118–129)	26.04.2016 (20.04.2016–02.05.2016)	0.9960	119.4

^a^The “duration” is the epidemic reporting duration (in days) since the starting time (date; day.month.year) of the reported outbreak, which is the difference of the end and start dates of the “epidemic reporting period”

^b^The “turning point” is the estimated time period (in days) from the starting time (date; day.month.year) of the outbreak to the estimated occurrence of the turning point

*Abbreviations*: AIC, Akaike information criterion; na, not applicable; this occurs when the fitting progress fails to converge for a few model frameworks; CI, confidence interval

**Fig. 3:**
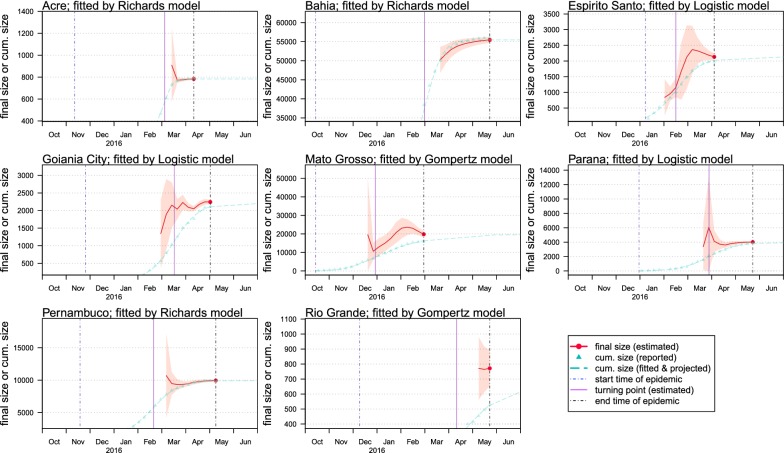
The estimation of final size (*K*) with variable turning points from the selected growth model. In each panel, the horizontal axis is the end time of fitting, and the vertical axis is the final size, *K*, or the reported number of cumulative (cum.) counts, *C*(*t*), of ZIKV incidences. The vertical dashed blue line indicates the start time of the epidemic, which is also the start time of fitting. The vertical dashed black line indicates the end time of the epidemic, which is also the largest end time of fitting. The vertical purple line is the estimated turning point, *τ*, by using the full dataset, which matches the models with the lowest AICs in Tables 1 and 2. The cyan curve is the fitted cumulative epidemic curve, and the triangular dots are the reported number of cumulative ZIKV incidences. The red line is the estimated final size against the end time of fitting. The red dot at the end is the final size estimation by using the full dataset, which matches the models with the lowest AICs in Tables 1 and 2. The red shading area represents the 95% CI

**Fig. 4:**
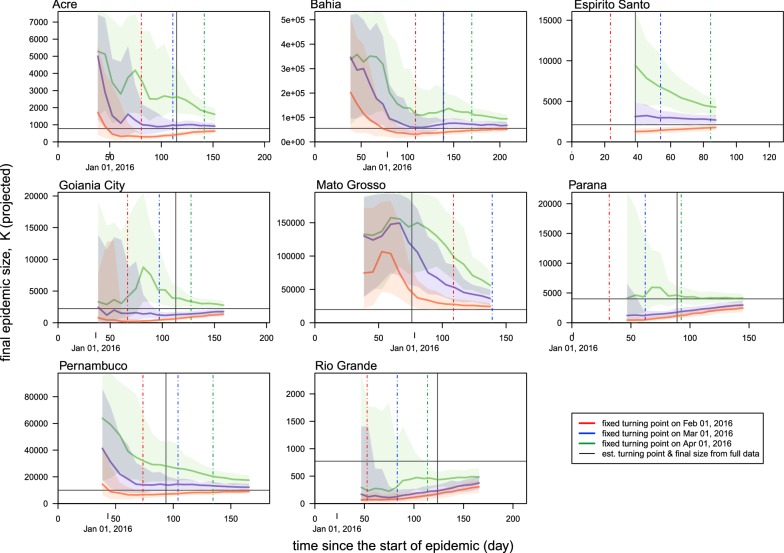
The estimation of final size (*K*) with fixed turning points. In each panel, the horizontal axis is the time since the start of the epidemic, which is also the end time (*T*_1_) of the dataset to train the growth model. The vertical axis is the projected final size, *K*. The vertical gray line is the estimated turning point, *τ*, by using the full dataset, which matches the models with the lowest AICs in Tables 1 and 2. The horizontal gray line is the estimated final size, *K*, by using the full dataset, which matches the models with the lowest AICs in Tables 1 and 2. The red curve is the real-time projection of *K* with *τ* fixed to be February 1, 2016 (vertical red dashed line). The blue curve is the real-time projection of *K* with *τ* fixed to be March 1, 2016 (vertical blue dashed line). The green curve is the real-time projection of *K* with *τ* fixed to be April 1, 2016 (vertical green dashed line). The shading area represents the 95% CI

To evaluate the potentials for the real-time estimation, we shortened the fitting period starting from the end time of the epidemic reporting period, and further checked the sensitivity of the estimates of *K* and *τ*. The epidemic reporting period is the period that local authority starts and ends the reporting of ZIKV cases, which is different from the real epidemic period. The real epidemic period starts earlier than the actual reporting starting date. For each state, the model with the lowest value of AIC is selected here, and all AIC values are summarized in Table 1 and Fig. 2. Table 2 summarizes the real-time estimation from the selected models. Figure 3 shows the relationship between the estimates of the epidemic size (or final size) and the end time of model fitting. We summarized the estimates using the incomplete dataset and using the complete dataset in Table 2, where the final epidemic size estimates by using the complete dataset match the red dots in Fig. 3. The early estimates of the turning points (*τ*) and reproduction numbers (*R*) are almost the same as the final results. The real-time estimates of epidemic size, *K*, converge to the estimates by using the full dataset (the red dots in Fig. 3), when the end time of the subsequent fitting period (*T*_*1*_) is longer than the turning point (*τ*), i.e. *T*_*1*_ > *τ*. The estimated epidemic sizes using the incomplete dataset are roughly consistent to the final estimates. Note that for a few states (e.g. Rio Grande), the estimated epidemic size is higher than the reported cumulative counts; this is due to the outbreak sustained after the end of disease notification (reporting) period. The epidemic size, *K*, is the final outbreak size until the end of the epidemic. Moreover, for all states we find that the epidemic sizes estimated 6–35 days after the turning points are indifferent from their final estimations, which means the 95% CIs are largely overlapping (Table 2). This finding indicates that the final outbreak size (*K*) can be estimated around the epidemic peaking stage by the projections from the simple growth models. By fixing *τ* to be the first days of February, March and April of 2016, the *K* projection converges as more data is including in the model training (Fig. 4). When the assumed turning point becomes closer to the real turning point, the projection of *K* will gain more accuracy and converges faster even during the early stage of the epidemics (i.e. before the occurrence of the real turning point).

**Table 2:** Summary table of the real-time estimation results from the selected models. The model with the lowest AIC (for the same states) is selected for analysis. The models results using the full epidemic dataset during the whole epidemic period match the models with the lowest AICs in Table 1. The numbers in parentheses are the 95% CIs

State	Model	Duration^a^	Fitting period	Final size	Reproduction number	Turning point^b^	Turning date
Acre	Richards	120	26.11.2015–26.03.2016	908 (576–1239)	2.16 (2.05–2.27)	113 (107–118)	18.03.2016 (13.03.2016–23.03.2016)
Acre	Richards	127	26.11.2015–02.04.2016	772 (747–797)	2.12 (2.05–2.18)	112 (110–114)	17.03.2016 (15.03.2016–19.03.2016)
Acre	Richards	134	26.11.2015–09.04.2016	776 (761–790)	2.12 (2.06–2.18)	112 (110–114)	17.03.2016 (15.03.2016–19.03.2016)
Acre	Richards	148	26.11.2015–23.04.2016	783 (774–793)	2.13 (2.07–2.19)	111 (110–113)	17.03.2016 (15.03.2016–18.03.2016)
Bahia	Richards	155	29.10.2015–02.04.2016	50,249 (46,852–53,646)	1.61 (1.57–1.65)	137 (131–143)	14.03.2016 (09.03.2016–20.03.2016)
Bahia	Richards	162	29.10.2015–09.04.2016	51,709 (49,237–54,181)	1.61 (1.57–1.66)	137 (132–141)	14.03.2016 (09.03.2016–19.03.2016)
Bahia	Richards	169	29.10.2015–16.04.2016	52,963 (50,927–55,000)	1.61 (1.57–1.66)	136 (132–141)	14.03.2016 (09.03.2016–18.03.2016)
Bahia	Richards	218	29.10.2015–04.06.2016	55,472 (54,683–56,260)	1.63 (1.58–1.68)	135 (132–139)	14.03.2016 (09.03.2016–16.03.2016)
Espirito Santo	Logistic	42	23.01.2016–05.03.2016	1671 (766–2577)	3.48 (2.22–5.30)	27 (9–46)	19.02.2016 (01.02.2016–08.03.2016)
Espirito Santo	Logistic	49	23.01.2016–12.03.2016	2126 (1112–3140)	2.93 (2.15–3.96)	35 (17–54)	27.02.2016 (09.02.2016–16.03.2016)
Espirito Santo	Logistic	56	23.01.2016–19.03.2016	2364 (1609–3119)	2.75 (2.21–3.40)	39 (26–53)	02.03.2016 (18.02.2016–15.03.2016)
Espirito Santo	Logistic	84	23.01.2016–16.04.2016	2132 (2020–2243)	3.05 (2.73–3.41)	35 (32–38)	27.02.2016 (24.02.2016–29.02.2016)
Goiania City	Logistic	113	10.12.2015–02.04.2016	2040 (1751–2329)	3.33 (3.05–3.63)	106 (102–111)	25.03.2016 (21.03.2016–30.03.2016)
Goiania City	Logistic	120	10.12.2015–09.04.2016	2230 (2015–2445)	3.19 (2.98–3.41)	109 (105–112)	28.03.2016 (25.03.2016–31.03.2016)
Goiania City	Logistic	127	10.12.2015–16.04.2016	2092 (1974–2210)	3.32 (3.12–3.53)	107 (105–109)	26.03.2016 (24.03.2016–28.03.2016)
Goiania City	Logistic	155	10.12.2015–14.05.2016	2243 (2184–2303)	3.07 (2.92–3.24)	109 (108–111)	29.03.2016 (27.03.2016–30.03.2016)
Mato Grosso	Gompertz	77	29.10.2015–14.01.2016	12,901 (8235–17,567)	2.03 (1.61–2.55)	58 (47–69)	26.12.2015 (16.12.2015–06.01.2016)
Mato Grosso	Gompertz	85	29.10.2015–23.01.2016	15,093 (10,750–19,436)	1.85 (1.57–2.18)	63 (53–73)	31.12.2015 (22.12.2015–10.01.2016)
Mato Grosso	Gompertz	92	29.10.2015–30.01.2016	17,550 (12,927–22,172)	1.72 (1.51–1.96)	68 (58–78)	05.01.2016 (26.12.2015–15.01.2016)
Mato Grosso	Gompertz	134	29.10.2015–12.03.2016	19,791 (18,147–21,435)	1.67 (1.56–1.79)	73 (69–76)	10.01.2016 (06.01.2016–14.01.2016)
Parana	Logistic	92	14.01.2016–16.04.2016	4121 (2604–5637)	2.90 (2.41–3.47)	86 (73–99)	09.04.2016 (27.03.2016–22.04.2016)
Parana	Logistic	99	14.01.2016–23.04.2016	3720 (3048–4393)	3.05 (2.63–3.53)	82 (75–89)	06.04.2016 (30.03.2016–13.04.2016)
Parana	Logistic	106	14.01.2016–30.04.2016	3610 (3228–3992)	3.12 (2.76–3.51)	81 (77–86)	05.04.2016 (31.03.2016–09.04.2016)
Parana	Logistic	141	14.01.2016–04.06.2016	4008 (3894–4123)	2.82 (2.66–2.99)	86 (84–87)	09.04.2016 (07.04.2016–11.04.2016)
Pernambuco	Richards	106	03.12.2015–19.03.2016	10,694 (4222–17,165)	1.81 (1.63–2.01)	94 (78–109)	06.03.2016 (19.02.2016–22.03.2016)
Pernambuco	Richards	113	03.12.2015–26.03.2016	9480 (7737–11,224)	1.78 (1.67–1.89)	91 (88–95)	04.03.2016 (29.02.2016–07.03.2016)
Pernambuco	Richards	120	03.12.2015–02.04.2016	9320 (8512–10,127)	1.77 (1.68–1.87)	91 (88–94)	03.03.2016 (29.02.2016–07.03.2016)
Pernambuco	Richards	169	03.12.2015–21.05.2016	9936 (9770–10,102)	1.85 (1.75–1.95)	90 (87–93)	02.03.2016 (29.02.2016–05.03.2016)
Rio Grande	Gompertz	148	24.12.2015–21.05.2016	771 (563–979)	1.54 (1.39–1.71)	120 (107–134)	22.04.2016 (09.04.2016–06.05.2016)
Rio Grande	Gompertz	155	24.12.2015–28.05.2016	765 (613–917)	1.54 (1.42–1.68)	120 (110–130)	22.04.2016 (12.04.2016–02.05.2016)
Rio Grande	Gompertz	162	24.12.2015–04.06.2016	772 (653–890)	1.54 (1.43–1.65)	120 (112–128)	22.04.2016 (14.04.2016–30.04.2016)

^a^The “duration” is the fitting duration (in days) since the starting time (date; day.month.year) for fitting, which is the difference of the end and start dates of the “fitting period”

^b^The “turning point” is the estimated time period (in days) from the starting time (date; day.month.year) of the outbreak to the estimated occurrence of the turning point

## Discussion

We used simple non-linear growth models to study the temporal patterns of ZIKV epidemics in eight Brazilian states. We showed that three simple growth models can be adapted to model the ZIKV outbreak, with the best *R*^2^ reaching 0.995. The estimated dates of the turning points varied from January to April of 2016 for the eight states. The difference in the turning points indicates spatial heterogeneity in the timing of the outbreak. We found that four out of the eight states (i.e. 50%) had a turning point in March 2016, which matches the epidemic peaking time of the whole of Brazil in 2016 [4, 25]. It is interesting to note that the earliest turning point was estimated as January 10 (95% CI: January 06–January 14), 2016 in Mato Grosso state, around which the epidemic started to be reported in the neighboring state of Parana in the epidemiological week (EW) 2 of 2016. We suspect that the reporting of this cluster of cases could be triggered by the turning point, which is in-line with the findings in [44]. The timing of the turning points, i.e. the duration from the epidemic reporting start to the turning point (the “turning point” column in Table 1), was also found to be remarkably different between each state. This is different from the previous work for the six archipelagos in French Polynesia [44], which could be due to the large differences in the ZIKV epidemic reporting periods of the states in Brazil. The local conditions, e.g. demographic factors, public health policies, seasonality including meteorological factors and other factors affecting mosquito activities, probably varied in different states. Hence, the growth structure of the epidemic curve could be affected, and thus the turning points are likely to appear heterogeneously across states. To further evaluate the timely efficiency of the local ZIKV notification, we checked if the turning point appeared in the latter half of the whole reporting period. This can be simply quantified by calculating the ratio of the “turning point” over “duration” in Table 1, and compared with 0.5. Most of the states had a turning point in the latter half of the outbreak reporting period except for the state of Espirito Santo. Espirito Santo had a turning point (*τ*) in the former half of the epidemic period (*T*) significantly, i.e. *τ* < *T*/2, which is different from other states, thus an outlier.

The disease infectivity is measured by the reproduction number, *R*, during an outbreak (Table 1). The estimated *R*-values are significantly less than 2 (with 95% CIs lower than 2) in the four states of Bahia, Mato Grosso, Pernambuco and the Rio Grande. The *R*s are significantly larger than 2 in the four states of Acre, Espirito Santo, Goiania City and Parana. In states of Bahia, Mato Grosso and Pernambuco, there were ZIKV cases confirmed since early 2015 [4]. Thus, the lower *R-*values are likely to be due to the depletion of susceptible population during the earlier outbreaks. In the state of Rio Grande, one possible explanation for the lowest *R* = 1.54 (95% CI: 1.43–1.65) is the relatively lower air temperature than most of the other places in Brazil. The average temperature starts to drop below 20 °C from March every year, during which the mosquito vector abundance is almost zero [65]. For the four states of Acre, Espirito Santo, Goiania City and Parana, ZIKV was not reported before October 2015 [4], and thus the *R-*values can also be treated as the estimates of the basic reproduction number, *R*_*0*_. Hence, we speculate that the *R*_*0*_ of ZIKV ranges from 2.07 to 3.41 by directly finding the range of the 95% CIs of *R-*values in states of Acre, Espirito Santo, Goiania City and Parana. The average value of *R*_*0*_ was 2.77. This average value and range of *R*_*0*_ is consistent with previous ZIKV studies for Brazil [4, 12, 14].

To evaluate the power for the real-time estimation, the selected models were repeatedly implemented with the fitting period starting from the end time of the reporting period, and thus we could further check the sensitivity of estimates of *K* and *τ* (Table 2). We report a converging real-time estimation of the final epidemic size starting on or after the turning date. The estimation of the turning points is obviously stable and consistent with the final estimation. Moreover, for all states, the epidemic sizes estimated 6–35 days after the turning points are virtually indifferent from their final estimations (i.e. estimates of *K* by using the full dataset). These findings reveal the real-time estimating potentials for the simple growth models proposed in this study. The final epidemic size (*K*) can be predicted at or after the peaking time of the epidemic. The early prediction of the final outbreak size (*K*) was found to depend on the timing of the turning point (*τ*), as shown in Fig. 3. Although projecting the temporal trends of an outbreak from the early-stage incomplete dataset could be sometimes misleading, we note that the predictions are reasonably accurate when the fitting dataset covers the turning point, which is in-line with the findings in [46, 66]. With data coming in from an ongoing outbreak, the performance of models used in this work will be continuously improved, thus real-time estimates of key epidemiological parameters may be available before the epidemic fully ends.

Since the prediction should be before the occurrence of an event, we note that the turning points forecast is difficult to be achieved with the simple models, which was also reported by previous Zika and dengue modelling literature [44, 45]. Nevertheless, we highlight the importance of a successful turning point forecast in the prediction of other epidemiological parameters. Our findings suggest that the projection on the epidemic final size (*K*) converges after using the data with time duration slightly over the turning point. In other words, once the knowledge of the turning point is equipped, the real-time estimation can be largely improved and converges quickly. To estimate the turning point (*τ*) we may not only rely on surveillance case data but also take into account of practical knowledge and factors that affect the disease transmission. For instance, the Zika fever in this work is a disease whose transmission depends largely on the activity of mosquitoes, which has strong seasonality. Local mosquito abundance drops to a sufficiently low level from May each year [64], which could largely reduce the ZIKV spread. Hence, *τ* is probably before May 2016. By fixing *τ* on the first day of February, March and April 2016, the *K* projection converges as more data is included in the training of the model (Fig. 4). When the assumed turning point approaches the real turning point, the projection of *K* will approach the estimate based on full data, and converge faster even during the early stage of the epidemics, i.e. before the arrival of the real turning point.

Besides the three models adopted in this study, there are other well-known non-linear growth models that have not been adopted. These unselected models include the four-parameter logistic, five-parameter logistic, Weibull and Sigmoid Emax models. One of the facts of the S-shape epidemic curve is that the growth starts from level zero. The Weibull and Sigmoid Emax models are more likely to yield inferior fitting performance with zero lower asymptote (or bound). Besides, the Sigmoid Emax model does not contain an intrinsic growth term. Thus, these two models are less popular in studying epidemic curves than the three models in Eqns (1–3). For the four-parameter logistic model, it is equivalent to the three-parameter version in Eqn (1) when the lower asymptote becomes zero. Although the five-parameter logistic model adds asymmetry factor (to control the asymmetry) based on the four-parameter version [67], it still contains the non-zero lower asymptote problem [68]. In addition, the five-parameter logistic model also could be over-sensitive for the early-stage prediction [46]. These shortcomings make it less practical than the Richards model in studying the epidemic curve. Therefore, we only adopt the three growth models in Eqns (1–3).

This work has some limitations. The analyses are highly reliant on the quality of the epidemic data, reporting delay and the change of reporting criteria. Since the local ZIKV surveillance are more reliable after the end of 2015 [26], we modeled the single-wave outbreaks on or after October 2015 and avoid including the dataset during early 2015. Due to the interference with the other *Flavivirus* (e.g. dengue virus, yellow fever virus, West Nile virus, etc.) [69], the serological diagnosis of ZIKV infection is less effective than the RT-PCR diagnosis. However, the time window for positive RT-PCR viremia is relatively short, roughly three to seven days, thus a suspected ZIKV should not be regarded as a negative case, which requires IgM tests for further confirmation [69]. Therefore, to avoid excluding the part of positive ZIKV cases in the suspected group, we considered the summation of suspected and confirmed cases as the incidence count for analysis. If the reporting delays, dates of onset, or the reporting rate are known, more realistic and comprehensive analysis can be performed that includes more accurate epidemic data and information. In this idealistic situation, although our simple non-linear models would be less attractive, they still could be used as the baseline framework for more advanced analysis, and to estimate the turning points.

## Conclusions

In this study, we analyzed the temporal patterns of epidemics in Brazil by using simple non-linear growth models. The average value of *R*_*0*_ was estimated to be 2.77 and varied from 2.07 to 3.41 in different states. We found spatial heterogeneity in the epidemiological features among the eight states. We propose a real-time estimation framework and we demonstrate that it is able to yield reliable real-time prediction of the final epidemic size. With precise knowledge of the turning point, the real-time projection of the final size is likely to be more accurate, even during the early stage of epidemics. Our modelling framework may be extended to study other infectious diseases epidemics, and easily implemented for a practical purpose.

## Additional file

**Additional file 1: Text S1.** The difference between the three growth models.

## Data Availability

All data used for analysis are freely available in the supplementary materials of Ferguson et al. [4].

## Abbreviations

ZIKV: Zika virus
CI: confidence interval
EW: epidemiological week
MGF: moment generating function
SD: standard deviation
AIC: Akaike information criterion
NLS: non-linear least squares
GBS: Guillain-Barré syndrome
