# Supplementary material for: Simple framework for real-time forecast in a data-limited situation: the Zika virus (ZIKV) outbreaks in Brazil from 2015 to 2016 as an example
Source: Parasit Vectors. 2019 Jul 12;12:344. doi: 10.1186/s13071-019-3602-9 (PMC6624944; doi:10.1186/s13071-019-3602-9)
Supplement: Supplementary file 1 — Additional file 1: Text S1. The difference between the three growth models. [file 13071_2019_3602_MOESM1_ESM.docx]

# **Additional file 1. Text S1 The difference between three growth models**

All the three models in Eqns (1)-(3) are actually the solutions of three differential equations, which can be written in a general form as follows.

$\frac{dC(t)}{dt}=\gamma C\cdot f(C)$. (A1)

Here, the term *γ* is the intrinsic per capita growth rate of the infected population. The difference between the three models is in the form of *f*(*C*).

For Logistic model in Eqn (1) of the main text, *f*(*C*) = 1 – *C*/*K*. The term *K* is the maximum cumulative incidence number of the epidemic, i.e., the final epidemic size.

For Gompertz growth model in Eqn (2) of the main text, *f*(*C*) = ln(*C*/*K*). The *f*(*C*) of Logical model is the first two terms of the Taylor expansion series of *f*(*C*) of the Gompertz model.

For Richard model, which is also called generalized logistic model, in Eqn (3) of the main text, *f*(*C*) = 1 – (*C*/*K*)*^α^*. Here, the term *α* is the exponent of deviation of the cumulative S-shaped epidemic curve. It can be seen that the logistic model is a special case of Richard model when *α* = 1.
